# Proteomic Studies Reveal Disrupted in Schizophrenia 1 as a Player in Both Neurodevelopment and Synaptic Function

**DOI:** 10.3390/ijms20010119

**Published:** 2018-12-29

**Authors:** Adriana Ramos, Carmen Rodríguez-Seoane, Isaac Rosa, Irantzu Gorroño-Etxebarria, Jana Alonso, Sonia Veiga, Carsten Korth, Robert M. Kypta, Ángel García, Jesús R. Requena

**Affiliations:** 1 CIMUS Biomedical Research Institute, University of Santiago de Compostela-IDIS, 15782 Santiago de Compostela, Spain; C.Rodriguez@ed.ac.uk (C.R.-S.); isaacrbenito@gmail.com (I.R.); sonia.veiga@usc.es (S.V.); angel.garcia@usc.es (Á.G.); jesus.requena@usc.es (J.R.R.); 2 Department of Psychiatry and Behavioral Sciences, Johns Hopkins University, Baltimore, MD 21287, USA; 3 Department of Pharmacology, University of Santiago de Compostela, 15782 Santiago de Compostela, Spain; 4 Cell Biology and Stem Cells Unit, CIC-bioGUNE, Parque Tecnologico de Bizkaia, 48160 Derio, Spain; igorrono@cicbiogune.es (I.G.-E.); rkypta@cicbiogune.es (R.M.K.); 5 Proteomics Unit, IDIS, 15706 Santiago de Compostela, Spain; janaalonsol@hotmail.com; 6 Department of Neuropathology, Heinrich Heine University, Medical School, 40225 Düsseldorf, Germany; ckorth@uni-duesseldorf.de; 7 Department of Surgery and Cancer, Imperial College London, London W12 OUQ, UK; 8 Department of Medical Sciences, University of Santiago de Compostela, 15782 Santiago de Compostela, Spain

**Keywords:** DISC1, neurodevelopment, synapse, CRMP-2, proteomics

## Abstract

A balanced chromosomal translocation disrupting DISC1 (Disrupted in Schizophrenia 1) gene has been linked to psychiatric diseases, such as major depression, bipolar disorder and schizophrenia. Since the discovery of this translocation, many studies have focused on understating the role of the truncated isoform of DISC1, hypothesizing that the gain of function of this protein could be behind the neurobiology of mental conditions, but not so many studies have focused in the mechanisms impaired due to its loss of function. For that reason, we performed an analysis on the cellular proteome of primary neurons in which DISC1 was knocked down with the goal of identifying relevant pathways directly affected by DISC1 loss of function. Using an unbiased proteomic approach, we found that the expression of 31 proteins related to neurodevelopment (e.g., CRMP-2, stathmin) and synaptic function (e.g., MUNC-18, NCS-1) is altered by DISC1 in primary mouse neurons. Hence, this study reinforces the idea that DISC1 is a unifying regulator of both neurodevelopment and synaptic function, thereby providing a link between these two key anatomical and cellular circuitries.

## 1. Introduction

The Disrupted in Schizophrenia 1 (DISC1) gene was found mutated when studying a chromosomal translocation t(1;11)(q42.1;q14.3) in a Scottish family; this translocation correlated with cases of schizophrenia, bipolar disorder and major depression [1,2]. Further studies also found that the truncation of this gene in an American family segregated with cases of schizophrenia [3]. 

Since the discovery of this translocation, many groups have invested their efforts in understanding the role of DISC1 protein, with the hope of revealing new mechanisms that could explain the neurobiology behind mental disease. Therefore, DISC1 was proposed to be involved in diverse processes such as neurogenesis [4,5], synapse regulation [6,7,8,9,10], neurite outgrowth [6,11,12], and neural migration and proliferation [13,14,15]. Also, yeast two hybrid experiments [16] and other molecular studies have revealed several important interacting partners of DISC1 including GSK3β [5], PDE4B [17], Rac1 [8], Girdin [18] or TNIK [9] among others. Thus, DISC1 might act as a molecular scaffold, providing cohesion and coordination among different biological events in the brain [19]. 

To acquire a deeper understanding of the mechanisms of action of DISC1, several proteomic analyses have been conducted to specifically address the role of the truncated isoform of DISC1 on the cellular proteome of neural cells [20,21]. In this study, we decided to specifically address the role of DISC1 loss of function, for that we carried out an unbiased proteomic analysis in DISC1-silenced neurons.

We report that DISC1 alters the expression of many relevant proteins related to neurodevelopment and synaptic function, reinforcing the idea that DISC1 is a key molecular link bridging neurodevelopmental functions with the regulation of synaptic formation and neurosignaling processes.

## 2. Results

### 2.1. Proteomic Analysis

Cell extracts from control and DISC1 knockdown murine primary neurons (Figure S1) were subjected to proteomic analysis. Four bidimensional gels for the silenced condition versus four of the control condition were analyzed. 3474 identical spots per gel were detected (Figure S2) and 75 of them were found differentially expressed with a fold change ≥2 and *p* value < 0.05 (Table S1). 68 of these spots were identified using mass spectrometry, corresponding to 48 unique proteins (Table 1). The functions of these proteins were mainly related to neurodevelopmental processes or synaptic function (Table 1, Figure S3). Particularly, 19 of them were related to neurodevelopmental processes (Table 1) and other 19 unique proteins were related to synaptic function (Table 1). Of note, 7 of these proteins have shared functions (Table 1, Figure S3). Therefore, these results suggest that DISC1 plays an important role linking these two processes.

Remarkably, some of the identified proteins have previously been described as DISC1 binding partners, it is the case of 14-3-3 proteins [12] and LIS1 [22], while CRMP-2 has been identified as a possible DISC1 interactor [16]. However, to the best of our knowledge, this is the first time that DISC1 has been found to also alter their expression. As well, we could identify some of the proteins as substrates of similar enzymes; this is the case of stathmin, CRMP-2, and MAP1B. These proteins are known to be phosphorylated by GSK3β to exert their functions. 

### 2.2. Ingenuity Pathway

To identify common molecular pathways regulated by DISC1 in our sample set we used the Ingenuity Pathways Analysis (IPA) software. The 5 top canonical pathways involved in our analysis are represented in Table 2. It is interesting that CRMP (collapsin response mediator protein) family was highlighted in the analysis as part of the Semaphorin signaling in neurons, since this signaling cascade is known to play an important role in neuronal differentiation and axonal growth [23,24]. Previous studies also concluded that the overexpression of the truncated isoform of DISC1 leads to dysregulation of Semaphorin signaling [20]. This could be a corroborative evidence for the fact that DISC1 expression has to be tightly and precisely regulated in a small window and that both, above and below that window you have dysregulation of similar signaling pathways.

The top molecular and cellular functions identified by IPA are represented in Table 3. The analysis particularly highlighted proteins involved in neurite outgrowth and branching of neurons. 

### 2.3. DISC1 Alters the Expression of Neurodevelopmental Related Proteins

Considering the results obtained by IPA analysis we focused on the collapsin response mediator proteins (CRMPs) to perform our validations. These proteins constitute a family of five homologous cytosolic proteins (CRMP-1-5) involved in microtubule regulation. All of them are phosphorylated and highly expressed in the developing and adult nervous system where they play important roles in neuronal development and maturation [25]. Six spots corresponding to CRMP-5, CRMP-3, CRMP-2 and CRMP-1 were differentially expressed in silenced vs. control cells (Table 1) in our study; in all cases the proteins were upregulated in DISC1 silenced cells. 

**Figure 1:**
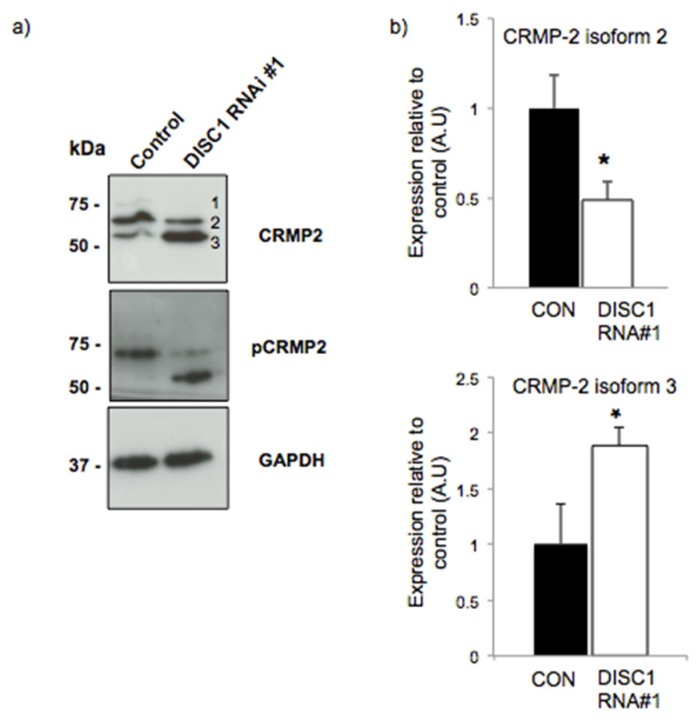
DISC1 differentially affects CRMP2 isoform levels. (**a**) Western blot of CRMP2 and pCRMP2 proteins. The total content of CRMP2 falls in DISC1 silenced cells, and the smallest one, thought to be a cleavage product, rises. The three isoforms are indicated (1–3). (**b**) Densitometric analysis of CRMP2 bands 2 and 3 (*n* = 4, * *p* < 0.05).

Particularly, CRMP-2 has been described as a candidate gene for susceptibility to schizophrenia [26] and was found upregulated in a proteomic study performed with brain samples from patients with bipolar disorder, schizophrenia and major depression [27]. We showed differential expression of multiple CRMP2 isoforms upon DISC1 silencing (Figure 1) in primary neurons. The existence of different isoforms of CRMP2 has been highlighted in several studies [28,29]. Here, CRMP2 was detected as three isoforms (labelled 1 to 3). Isoforms 1 and 2, most likely corresponding to CRMP2A and CRMP2B [28] were found to be downregulated in DISC1-silenced cells, while isoform 3 was upregulated. A similar pattern was observed using antibodies that recognize CRMP-2 phosphorylated at Thr-514 (Figure 1). Therefore, isoform 3 most likely corresponds to the spot that was differentially expressed in our proteomic analysis.

Some studies described this isoform as a calpain-associated degradation product [30,31], while others highlight its role in neurite outgrowth inhibition [32]. If this is the case, it suggests that DISC1 silencing leads to increased expression of CRMP-2 and, as a result, inhibition of neurite outgrowth. Of note, Septin-5, a protein that directly interacts with CRMP-2, was also found differentially expressed in our study (Table 1). 

### 2.4. DISC1 Alters the Expression of Synaptic Function Related Proteins

We also consider of great relevance that endocytosis was highlighted under the top molecular and cellular functions in our IPA analysis (Table 3). Endocytosis and exocytosis are crucial processes for neurotransmission [33] and regulated by SNARE and SM proteins (Sec1/Munc18-like proteins) [34]. In particular, syntaxin-7 (member of the SNARE complex present on plasma membrane) and syntaxin binding protein (STXBP, also known as MUNC18) were found upregulated in DISC1-silenced cells (Table 1). Other proteins that regulate the exocytic processes responsible for neuronal communication are Rab proteins [35], which catalyze SNARE complex assembly [36]. In this study four different Rab proteins were found differentially expressed in DISC1-silenced cells (Table 1).

### 2.5. DISC1 Silenced SH-SY5Y Cells Show Impaired Neurite Outgrowth

**Figure 2:**
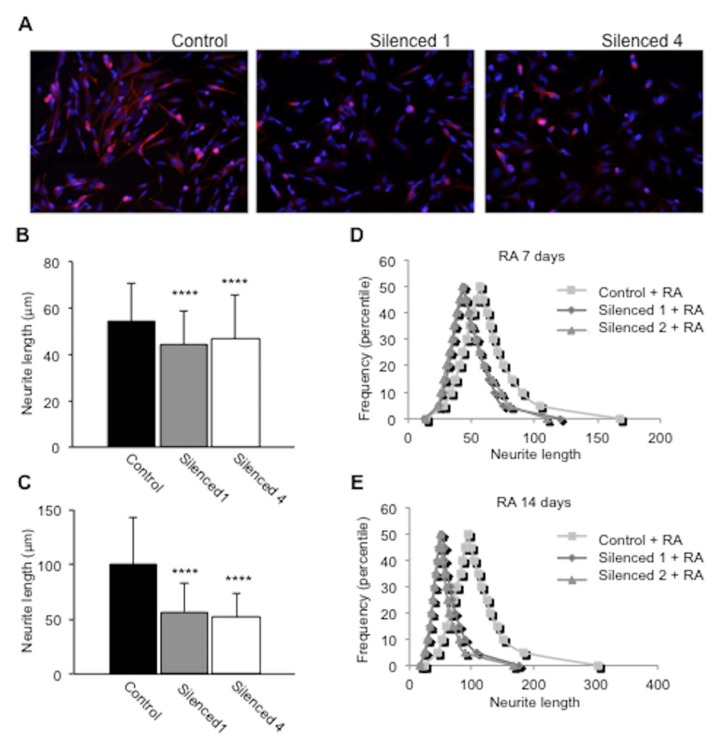
DISC1-silenced cells show morphological impairment in neurite outgrowth assays. Cells were treated with retinoic acid (RA) for 7 and 14 days and neurite length was measured using Image J. (**A**) Fluorescence images of SH-SY5Y cells expressing control and DISC1 shRNAs treated with RA for 7 days and immunostained for βIII-tubulin (red); nuclei were stained using DAPI (blue). (**B**,**C**) Average neurite length ± SD; (**** *p* < 0.0001, significantly different between control and DISC1-silenced cells, *n* > 200 for each cell line). (**D**,**E**) Frequency (percentile) of cells according to neurite length at 7 days (**D**) and 14 days (**E**) for each cell population; *p* < 0.0001 control vs. silenced 1 at 7 and 14 days, *p* < 0.0001 control vs. silenced 4 at 7 days, *p* < 0.001 control vs. silenced 4 at 14 days (Mann–Whitney U test).

To further test that silencing of DISC1 results in disruption of neural development, we performed a morphological study in SH-SY5Y cells in which DISC1 was silenced [37]. The absence of DISC1 in this cell line resulted in morphological changes (Figure 2). Thus, upon retinoic acid-induced differentiation, DISC1-silenced cells exhibited fewer and shorter neurites (Figure 2, Figure S4). 

## 3. Discussion

We have taken advantage of a well-established murine primary neuron DISC1 knock-down experimental system [8,14,37] to carry out an unbiased proteomic analysis and thus, identify proteins which have their expression affected by DISC1. 

The results of our analysis highlight the importance of DISC1 both in neurodevelopment and synaptic regulation. Both functions have been already ascribed to DISC1; however, this study describes new important routes to explore, as the effect DISC1 silencing on the expression of CRMP family of proteins. This could be a powerful mechanism to further investigate considering the relevance this family of proteins has in the neurobiology of mental disease [27,38,39,40].

Furthermore, DISC1 knockdown resulted in a neurite outgrowth deficit in RA-treated SH-SY5Y cells. Previous studies have reported an impaired neurite outgrowth in cell models that overexpress mutant isoforms of DISC1 [11,41] and an increase of neurite outgrowth was seen in PC12 cells that overexpress DISC1 [42]. Therefore, our study reinforces the idea that the loss of function of DISC1 is critical for proper regulation of neurite outgrowth. In this direction, other studies have previously shown DISC1 silencing affected neurite outgrowth using PC12 cells [14]. We have to consider neurite outgrowth in PC12 cells is a result of two processes, neural differentiation and subsequent neurite extension, so the effects of silencing may be interpreted as measuring an effect on either/both processes. In contrast, SH-SY5Y cells are already neuronal and forming neurites, so we could compare neurite length and the effect is specific to neurite outgrowth.

At the same time, we have found that several proteins that participate in synaptic membrane trafficking and synapse formation are altered in DISC1 silenced neurons, such as syntaxin 7, MUNC-18, cadherin-13, and Rab proteins (Table 1), but we cannot conclude whether trafficking is up- or downregulated in our system. Previous studies have shown that DISC1 enhances the transport of synaptic vesicles, therefore we could expect that knocking down DISC1 expression produced an attenuated vesicle transport in primary cortical neurons [43]. 

Summarizing, our study shows that DISC1 works as an important modulator of proteins that are directly involved both in neurodevelopment and in adult synaptic regulation, representing a unifier factor of two seemingly different categories.

## 4. Materials and Methods

### 4.1. Antibodies

Commercial antibodies specific for the following proteins were used: CRMP-2, p(Thr514)CRMP-2, Stathmin, p(Ser38)Stathmin (1:1000; Cell Signaling Technology, Danvers, MA, USA); tubulin, GAPDH (1:5000; Sigma-Aldrich, St. Louis, MO, USA); the human DISC1-specific antibody 14F2 has been previously described [44]; the mouse DISC1-specific antibody D27 was a kind gift from Merck (Kenilworth, NJ, USA). Goat anti-rabbit (1:2000; Dako Cytomation, Glosstrup, Denmark), sheep anti-mouse (1:5000; GE Healthcare Amersham Bioscience, Uppsala, Sweden) and donkey anti-goat (1:2000; Santa Cruz Biotechnologies, Santa Cruz, CA, USA) were used as secondary antibodies. 

### 4.2. Cell Culture

SH-SY5Y neuroblastoma cells (European Collection of Cell Cultures, Salisbury, UK) were maintained in 1:1 Earle’s Balanced Salt Solution (EBSS)- F12HAM (Sigma Aldrich) with 15% fetal bovine serum (FBS) (Gibco, Life Technologies, Gaithesburg, MD, USA), 1% Glutamine (Gln) (Sigma Aldrich), 1% non-essential amino acids (NEAA) (Sigma Aldrich), and 1% Penicillin-Streptomycin (P/S) (Invitrogen). 293FT cells (Invitrogen) were maintained in Dulbecco’s Modified Eagle’s Medium (DMEM) (Sigma Aldrich) with 10% FBS, 1% sodium pyruvate (Sigma Aldrich), 1% NEAA, 1% Gln, and 1% P/S. 

Murine cortex and hippocampal primary neurons were prepared from 14–15 days embryos (see below ethical statement). Pregnant dams were killed by cervical dislocation in accordance with institutional guidelines for care and use of animals. The embryos were maintained and dissected in PBS Ca/Mg (Invitrogen) supplemented with 33 mM glucose. Pooled tissue was mechanically dissociated, treated with trypsin (Invitrogen) and DNaseI (Roche Applied Science, Mannheim, Germany) and resuspended in Neurobasal medium (Invitrogen) supplemented with 50X B27 (Invitrogen), 0.55g/100mL glucose (Sigma Aldrich), 42 mg/100 mL sodium bicarbonate (Sigma Aldrich), 1% P/S and 1% glutamine. The cells were plated on poly-D-lysine (Sigma Aldrich) coated Petri dishes. Cultures were maintained in serum free medium at 37 °C in 95% air/5% CO_2_.

### 4.3. Ethics Statement

Animal experiments were carried out in accordance with the European Union Council Directive 86/609/EEC, and were approved by the University of Santiago de Compostela Ethics Committee (protocol 15005AE/12/FUN 01/PAT 05/JRR2, 5 January 2012).

### 4.4. DISC1 Silencing

For DISC1 knock-down in murine primary neurons, we chose a validated shRNA construct developed by Akira Sawa’s group (DISC1 RNAi #1) that has been shown to specifically decrease the amount of DISC1 in cortical neural cell cultures [8,14,37]. The commercial pLK0.1-puro non-mammalian shRNA control construct from Sigma Aldrich (reference: SHC002) was used as a scramble control. Lentiviruses were produced by calcium phosphate triple co-transfection of shRNA (see Table S3 and Figure S1 in Supporting Information), VSVG and ΔR8.9 constructs into 293FT packaging cells. Virus-containing medium was collected 48 h after transfection, and added (10 mL of lentiviral solution/3 × 10^6^ neurons) to the medium of primary neurons at 7 DIV. The medium was changed 24 h after infection, and incubation continued for 72 h.

In SH-SY5Y cells, DISC1 was silenced using commercial Mission^®^ shRNA lentiviral transduction particles (Sigma Aldrich, reference NM_018662) containing two alternative PLKO.1-Puro-CMV shRNA plasmids (Table S2 in Supporting Information). Mission^®^ pLKO.1-puro non-mammalian shRNA particles (reference: SHC002V) were used as control. Stable cell lines were generated for any of these constructs after selection with puromycin as previously described [37]. 

### 4.5. Sample Preparation for Proteomic Studies

Cells (confluent 100 mm plates) were washed twice with cold PBS and solubilized in lysis buffer (20 mM HEPES, 2 mM EGTA, 1 mM DTT, 1 mM sodium orthovanadate, 1% Triton X-100, 10% Glycerol, 2 µM leupeptin, 400 µM PMSF, 50 µM β-glycerophosphate, 100 µg/mL Trasylol). The cells were scraped on ice for 10 min, incubated on ice for 30 min with periodic vortexing every 5 min and centrifuged for 20 min at 14,000 *g*, 4 °C. The supernatant was saved and the pellet discarded. The protein content was determined using the BCA protein assay kit (Pierce Chemical). Proteins were precipitated with 60% trichloroacetic acid (TCA) in acetone. After 2–3 acetone washes, proteins were dissolved in 500 μL of 2D sample buffer (5 M urea, 2 M thiourea, 2 mM tributyl-phosphine, 65 mM DTT, 65 mM CHAPS, 0.15 M NDSB-256, 1 mM sodium vanadate, 0.1 mM sodium fluoride, and 1 mM benzamidine). Ampholytes (Servalyte 4–7) were added to the sample to a final concentration of 1.6% (*v*/*v*). 

### 4.6. Proteomic Studies

The primary neuron cell lysates were subjected to two-dimensional gel electrophoresis (2-DE). Protein quantitation was performed with the Coomassie plus protein reagent (Thermo Scientific, Asheville, NC). Five hundred micrograms of protein were loaded onto each gel to allow detection of low abundance proteins. Four gels per study group (DISC1 knock-down and control) were compared. Immobilized pH gradient (IPG) strips (4–7, 24 cm, GE Healthcare, Uppsala, Sweden) were rehydrated in the sample, and isoelectric focusing (IEF) was performed in a Multiphor (GE Healthcare) for 85 kVh at 17 °C. Following focusing, the IPG strips were immediately equilibrated for 15 min in 4 M urea, 2 M thiourea, 130 mM DTT, 50 mM Tris pH 6.8, 2% *w*/*v* SDS, 30% *v*/*v* glycerol. Later, the strips were placed for 15 min in the same buffer, in which DTT was replaced by 4.5% iodoacetamide (Sigma Aldrich). The IPG strips were placed on top of the second dimension gels and embedded with 0.5% melted agarose. Proteins were separated in the second dimension by SDS-polyacrylamide gel electrophoresis (PAGE) on 10% gels at run conditions of 10 °C, 20 mA per gel for 1 h, followed by 40 mA per gel for 4 h by using an Ettan Dalt 6 system (GE Healthcare). Following electrophoresis, gels were fixed in 10% methanol/7% acetic acid for 1 h, and stained overnight with Sypro Ruby fluorescent dye (Lonza, Switzerland). After staining, gels were washed for 1 h in 10% methanol/7% acetic acid, and scanned in a Typhoon 9410 (GE Healthcare).

### 4.7. Differential Image Analysis

Image analysis was performed with the Ludesi REDFIN 3 Solo software (Ludesi, Malmö, Sweden). The integrated intensity of each of the spots was measured, and the background corrected and normalized. Differential expression of proteins was defined on the basis of ≥2-fold change between group averages and *p* < 0.05.

### 4.8. Mass Spectrometric Analysis

Spots of interest were carefully excised and subjected to in-gel digestion with trypsin [45]. Tryptic digests were analyzed using a 4800 MALDI-TOF/TOF analyzer (Applied Biosystems). Dried peptides were dissolved in 4 µL of 0.5% formic acid. Equal volumes (0.5 µL) of peptide and matrix solution, consisting of 3 mg alpha-cyano-4-hydroxycinnamic acid (α-CHCA) dissolved in 1 mL of 50% acetonitrile in 0.1% trifluoroacetic acid, were deposited using the thin layer method, onto a 384 Opti-TOF MALDI plate (Applied Biosystems). MS spectra were acquired in reflectron positive-ion mode with a Nd:YAG, 355 nm wavelength laser, averaging 1000 laser shots and using at least three trypsin autolysis peaks as internal calibration. All MS/MS spectra were performed by selecting the precursors with a relative resolution of 300 (FWHM) and metastable suppression. Automated analysis of mass data was achieved by using the 4000 Series Explorer Software V3.5. MS and MS/MS spectra data were combined through the GPS Explorer Software v3.6. Database search was performed with the Mascot v2.1 search tool (Matrix Science, London, UK) screening SwissProt (release 56.0). Searches were restricted to mouse taxonomy allowing carbamidomethyl cysteine as a fixed modification and oxidized methionine as potential variable modification. Both the precursor mass tolerance and the MS/MS tolerance were set at 30 ppm and 0.35 Da, respectively, allowing 1 missed tryptic cleavage site. All spectra and database results were manually inspected in detail using the above software. Protein scores greater than 56 were accepted as statistically significant (*p* < 0.05), considering positive the identification when protein score CI (confidence interval) was above 98%. In case of MS/MS spectra, total ion score CI was above 95%.

### 4.9. SDS-PAGE and Western Blotting

A total of 50 µg of protein was mixed with Laemmli sample buffer (BioRad), heated at 100 °C for 10 min, spun, and the supernatant loaded on a 7.5% SDS-PAGE gel. Samples were subjected to electrophoresis and transferred to polyvinylidenedifluoride (PVDF) membranes (Millipore, Bedford, MA, USA). The conditions of the electrophoresis were 200 V, 1 h. Electrophoresis was performed using a Mini-PROTEAN 3 cell electrophoresis system (BioRad). The transfer was performed in a Trans-blot SD semi-dry transfer cell (BioRad) using the following conditions: 0.8 mA/cm^2^, 90 min. The PVDF membranes were blocked in 5% non-fat milk in PBS-0.1% Tween solution overnight at 4 °C, then 4 washes of 5 min with PBS-0.1% Tween20 were performed, and the membrane was incubated with the primary antiserum (in 5% BSA in PBS-0.1%Tween20) for 1 h at room temperature, washed again and incubated with the peroxidase-conjugated secondary antibody (in PBS-0.1% Tween20), and subjected to 4 washes of 5 min each with PBS-0.1% Tween20. Finally the membrane was incubated with the chemiluminescence solution Luminata Forte Western HRP substrate (Merck Millipore). To develop the membranes Hypercassette (GE Healthcare) and Amersham Hyperfilm ECL (GE Healthcare) were used. 

### 4.10. Ingenuity Pathway

Ingenuity Pathway Analysis software (Ingenuity Systems, CA, USA) was used to investigate interactions between all the 48 identified proteins. Interactive pathways were generated to observe potential direct and indirect relations among the differentially expressed proteins. To test the enriched pathways we consider as settings direct and indirect relationships that were experimentally observed.

### 4.11. Neurite Outgrowth Assays

Stable SH-SY5Y cell lines generated using TRCN0000118997 (Silenced 1), TRCN0000119000 (Silenced 4) and non-target shRNAs were cultured for 7 and 14 days in medium containing 10 µM retinoic acid (RA) (Sigma Aldrich). To analyze neurite outgrowth, images of live cells were taken under a microscope and processed using Image J software (http://rsb.info.nih.gov/ij). Cells with and without neurites longer than two cell bodies were counted in photomicrographs of the differentiated control and DISC1-silenced cells.

### 4.12. Immunocytochemistry of SH-SY5Y Cells

Retinoic acid-treated cells were fixed in paraformaldehyde and immunostained for β3-tubulin and nuclei were visualized using DAPI, as previously described by the authors of [46].

### 4.13. Statistical Analysis

One-way ANOVA was employed in the proteomic analysis to determine statistically significant differences between groups of samples. For each spot ID, ANOVA *p*-value was calculated using the quantified and normalized spots volumes for the matched spot in each of the images. Differential expression of proteins was defined on the basis of ≥2-fold change between group averages and *p* < 0.05.

In the neurite outgrowth assay, three fields of up to 100 cells were analyzed for each condition and the experiment was performed twice. Statistical analysis was performed using a non-parametric unpaired Mann-Whitney U-test (two-tailed); results were considered significant with *p* < 0.05.

## 5. Conclusions

This study shows DISC1 disrupts the expression of a number of proteins involved in neurodevelopment and synaptic function. Thus, DISC1 acts as a key modulator of two mechanisms that have been critically implicated in the development of mental disease.

## Figures and Tables

**Table 1:** Proteins involved in neurodevelopment or synaptic function identified through proteomic analysis of primary neurons ^1^.

Function	Protein	Fold Change	*p* Value
Neurite outgrowth or neural migration	Dihydropyrimidinase-related protein 5 (CRMP-5)	2.59	3.066 × 10^−5^
	Dihydropyrimidinase-related protein 3 (CRMP-3)	3.12, 2.23	1.469 × 10^−4^, 2.894 × 10^−4^
	Dihydropyrimidinase-relatedprotein 2 (CRMP-2)	2.21, 2.03	2.457 × 10^−4^, 0.059
	Dihydropyrimidinase-related protein 1 (CRMP-1)	2.10	9.180 × 10^−5^
	Tubulin alpha-1A chain (TBA1A)	2.01,2.99,2.13	0.0043, 1.326 × 10^−4^, 4.067 × 10^−4^
	Tubulin beta-2B chain (TBB2B)	Inf, 2.42	0.0065, 0.0156
	Microtubule-associated protein (MAP1B)	2.04, 2.20,3.03	3.661 × 10^−7^, 2.894 × 10^−4^, 2.717 × 10^−5^
	14-3-3 protein epsilon (14-3-3ε)	2.67, 3.19	6.713 × 10^−5^, 1.854 × 10^−4^
	14-3-3 protein zeta/delta (14-3-3θ/Δ)	8.42, 2.63, 3.26, 3.88, 3.89, 6.81, 3.82, 6.20	3.028 × 10^−6^, 2.334 × 10^−4^, 1.579 × 10^−4^ 9.307 × 10^−4^, 0.021, 2.080 × 10^−5^, 0.0022, 2.572 × 10^−6^
	14-3-3 protein gamma (14-3-3γ)	3.24, 2.30	6.104 × 10^−5^, 0.0084
	Platelet-activating factor acetylhydrolase IB (Lis-1)	2.48	0.0049
	Stathmin (STMN)	2.12, 4.64	8.206 × 10^−4^, 1.021 × 10^−4^
	Syntaxin-7 (STX7)	2.13	0.0022
	Tropomyosin alpha-3 chain (TPM3)	2.88	0.0115
	Actin, cytoplasmic 2 (ACTG)	4.94, 2.95	4.398 × 10^−5^, 1.081 × 10^−5^
	Cadherin-13 (CAD13)	2.31	0.0108
	Calreticulin (CALR)	2.60	0.0088
	Septin-5 (SEPT5)	2.15	0.0034
	Apolipoprotein A-I (APOA1)	2.41	9.215 × 10^−5^
	Dynamin 1 (DYN1)	4.33	1.440 × 10^−4^
Dynamin 1 (DYN1)	Dihydropyrimidinase-related protein 5 (CRMP-5)	2.59	3.066 × 10^−5^
	Dihydropyrimidinase-relatedprotein 2 (CRMP-2)	2.21, 2.03	2.457 × 10^−4^, 0.059
	Microtubule-associated protein (MAP1B)	2.04, 2.20, 3.03	3.661 × 10^−7^, 2.894 × 10^−4^, 2.717 × 10^−5^
	Transitional endoplasmic Reticulum ATPase (TERA)	2.21	5.537 × 10^−4^
	Stathmin (STMN)	2.12, 4.64	8.206 × 10^−4^, 1.021 × 10^−4^
	Syntaxin-binding protein 1 (STXB1)	3.43	0.0010
	Syntaxin-7 (STX7)	2.13	0.0022
	Ras-related protein Rab-1A (RAB1A)	2.01	0.0023
	Ras-related protein Rab-2A (RAB2A)	2.43	4.164 × 10^−4^
	Ras-related protein Rab-11B (RB11B)	3.25	0.0305
	Ras-related protein Rab-18 (RAB18)	3.23	5.527 × 10^−4^
	Cadherin-13 (CAD13)	2.31	0.0108
	Rho GDP-dissociation inhibitor 2 (GDIR2)	2.28	1.061 × 10^−4^
	Phosphatidylethanolamine-binding protein 1 (HCNP)	3.84, 6.49	4.081 × 10^−4^, 2.377 × 10^−4^
	Calreticulin (CALR)	2.60	0.0088
	Adaptin ear-binding coat-associated protein 1 (NECP1)	2.51	6.028 × 10^−5^
	Neuronal calcium sensor 1 (NCS1)	2.24	9.215 × 10^−5^
	Dynamin 1 (DYN1)	4.33	1.440 × 10^−4^

^1^ All the proteins had a fold change > 2 and *p* value < 0.05. Fold change in red indicates that the protein is overexpressed in DISC1 silenced cells, while fold change in black indicates a downregulation in DISC1 silenced cells.

**Table 2:** Ingenuity top canonical pathways.

Name	*p* Value	Proteins
14-3-3 mediated signaling	4.99 × 10^−7^	TUBA1A, 14-3-3G, TUBB2B, PDIA3,1 4-3-3E, 14-3-3Z
Semaphorin signaling in neurons	5.28 × 10^−6^	CRMP3, CRMP1, CRMP2, CRMP5
Remodeling of epithelial adherent junctions	1.52 × 10^−5^	DNM1L, TUBA1A, ACTG1, TUBB2B
Cell cycle: G2/M DNA damage checkpoint regulation	1.75 × 10^−4^	14-3-3G, 14-3-3E, 14-3-3Z
PI3K/AKT signaling	1.87 × 10^−4^	14-3-3G, 14-3-3E, HSP90AA1, 14-3-3Z

**Table 3:** Ingenuity Top 10 molecular and cellular functions.

Name	*p* Value	Proteins
Outgrowth of cells	3.94 × 10^−8^	DNM1L, TUBA1A, HBA1/HBA2, CRMP3, MAP1B, SET, PDIA3, CRMP2, 14-3-3G, HSP90AA1, CRMP5
Patterning of dendrites	9.56 × 10^−8^	CRMP1, CRMP2, GDA
Outgrowth of neurites	1.94 × 10^−7^	DNM1L, TUBA1A, HBA1/HBA2, DPYSL3, MAP1B, SET, PDIA3, CRMP2, 14-3-3Z, CRMP5
Branching of neurons	2.53 × 10^−7^	DNM1L, HNRNPK, CRMP3, MAP1B, PDIA3, CRMP1, CRMP2, CRMP5, GDA
Organization of cytoplasm	7.08 × 10^−7^	CDH13, RAB2A, HNRNPK, CRMP1, CRMP2, CRMP5, STMN1, CALR, TPM3, DNM1L, ACTG1, PEX5, CRMP3, MAP1B, RAB1A, PDIA3, HSP90AA1, GDA
Fibrogenesis	8.53 × 10^−7^	CALR, CDH13, TPM3, ACTG1, CRMP3, MAP1B, APOA1, CRMP2, GDA, STMN1
Endocytosis	1.39 × 10^−6^	CALR, CDH13, HNRNPK, MAP1B, RAB1A, APOA1, CRMP2, VCP, HSP90AA1, NECAP1
Neuritogenesis	2.09 × 10^−6^	DNM1L, HNRNPK, CRMP3, MAP1B, PDIA3, CRMP1, CRMP2, HSP90AA1, CRMP5, GDA, STMN1
Branching of neurites	2.50 × 10^−6^	DNM1L, HNRNPK, MAP1B, PDIA3, CRMP1, CRMP2, CRMP5, GDA
Microtubule dynamics	3.39 × 10^−6^	CDH13, RAB2A, HNRNPK, CRMP1, CRMP2, CRMP5, STMN1, TPM3, DNM1L, ACTG1, CRMP3, MAP1B, PDIA3, HSP90AA1, GDA

## Supplementary Materials

Supplementary materials can be found at http://www.mdpi.com/1422-0067/20/1/119/s1.

## Abbreviations

2-DE	Two-dimensional electrophoresis
CRMP-2	Collapsing response mediator protein 2
DISC1	Disrupted in Schizophrenia 1
MAP1B	Microtubule associated binding protein 1
MUNC18	Mammalian uncoordinated-18
MALDI	Matrix-assisted laser desorption ionization
MS	Mass Spectrometry
NCS-1	Neural calcium sensor 1
SM	Sec1/Munc18-like proteins
SNARE	(Soluble NSF Attachment Protein) Receptor
WB	Western blot
