# Supplementary material for: Proteomic Studies Reveal Disrupted in Schizophrenia 1 as a Player in Both Neurodevelopment and Synaptic Function
Source: Int J Mol Sci. 2018 Dec 29;20(1):119. doi: 10.3390/ijms20010119 (PMC6337115; doi:10.3390/ijms20010119)
Supplement: Supplementary file 1 [file ijms-20-00119-s001.zip › supplementary/ijms-400252-suppl..pdf]

Supporting information

Real-time PCR

RNA was extracted from cell homogenates using TRIzol (Invitrogen). Removal of residual DNA was performed with RNase free DNase (Roche Applied Science). cDNA (20 µl) was prepared from 1 µg of RNA using random primers and M-MLV reverse transcriptase (Invitrogen). One microliter of the cDNA solution was then used together with gene specific primers (Supplementary Table 4) and the Light Cycler TaqMan Master kit (Roche Applied Science) following the manufacturer´s protocol. The housekeeping gene RNApolII was used as an endogenous control. Real-time quantitative PCR was performed using a Light Cycler 2.0 sequence detection system with 26 capillary format. PCR cycling conditions were: 95ºC for 15 minutes, 40 cycles of 95ºC for 20 seconds and 60ºC for 1 minute. PCR data were obtained with the Light Cycler Probe design software version 4.1 (Roche Applied Science) and quantified by a standard curve method (Livak & Schmittgen 2001).

References

Livak, K. J. and Schmittgen, T. D. (2001) Analysis of relative gene expression data using real-time quantitative PCR and the 2(-Delta Delta C(T)) Method. *Methods,* **25,** 402-408.

Supplementary Figure 1: DISC1 knockdown expression in neuron primary cell culture. Western blot analysis and densitometric analysis. ***$p<0.001$ using paired t test.

Supplementary Figure 2: Representative gel obtained during the proteomic analysis. Identified spots with a fold-change >2 appear highlighted with their mass spectrometry identification number. a) Bidimensional gel image b) Zoom of the lower left corner of the gel.

Supplementary Figure 3: Classification of the proteins identified in the proteomic study according to their functions.

Supplementary Figure 4: DISC1-silenced cells have altered morphological and reduced neurite outgrowth. Representative bright field images for control and DISC1-silenced stable cell lines treated with retinoic acid (RA) for 7 days.

Supporting Tables

Table S2: shRNAs used to silence DISC1 in SH-SY5Y cells

NAME	shRNA commercial REFERENCE	SEQUENCE
SILENCED 1	TRCN0000118997	5'-CCGAGGAGATTAGATCATTAAC-3'
SILENCED 4	TRCN0000119000	5'-CGATTGCTTATCCAG-3'

Table S3: shRNA used to silence DISC1 in neurons

shRNA	SEQUENCE
DISC1 shRNA#1	5'-GGCAAACACTGTGAAGTGC-3'

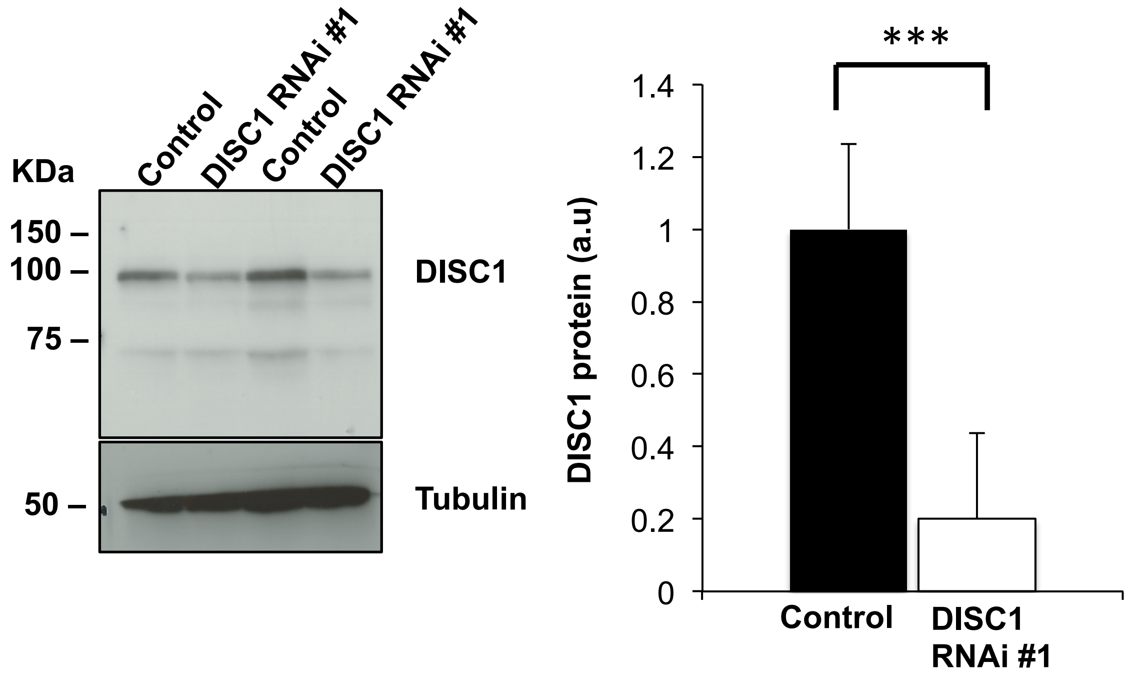
KDa
Control
DISC1 RNAi #1
Control
DISC1 RNAi #1
150 –
100 –
75 –
50 –
DISC1
Tubulin
***
DISC1 protein (a.u)
1.4
1.2
1
0.8
0.6
0.4
0.2
0
Control
DISC1
RNAi #1

Figure S1

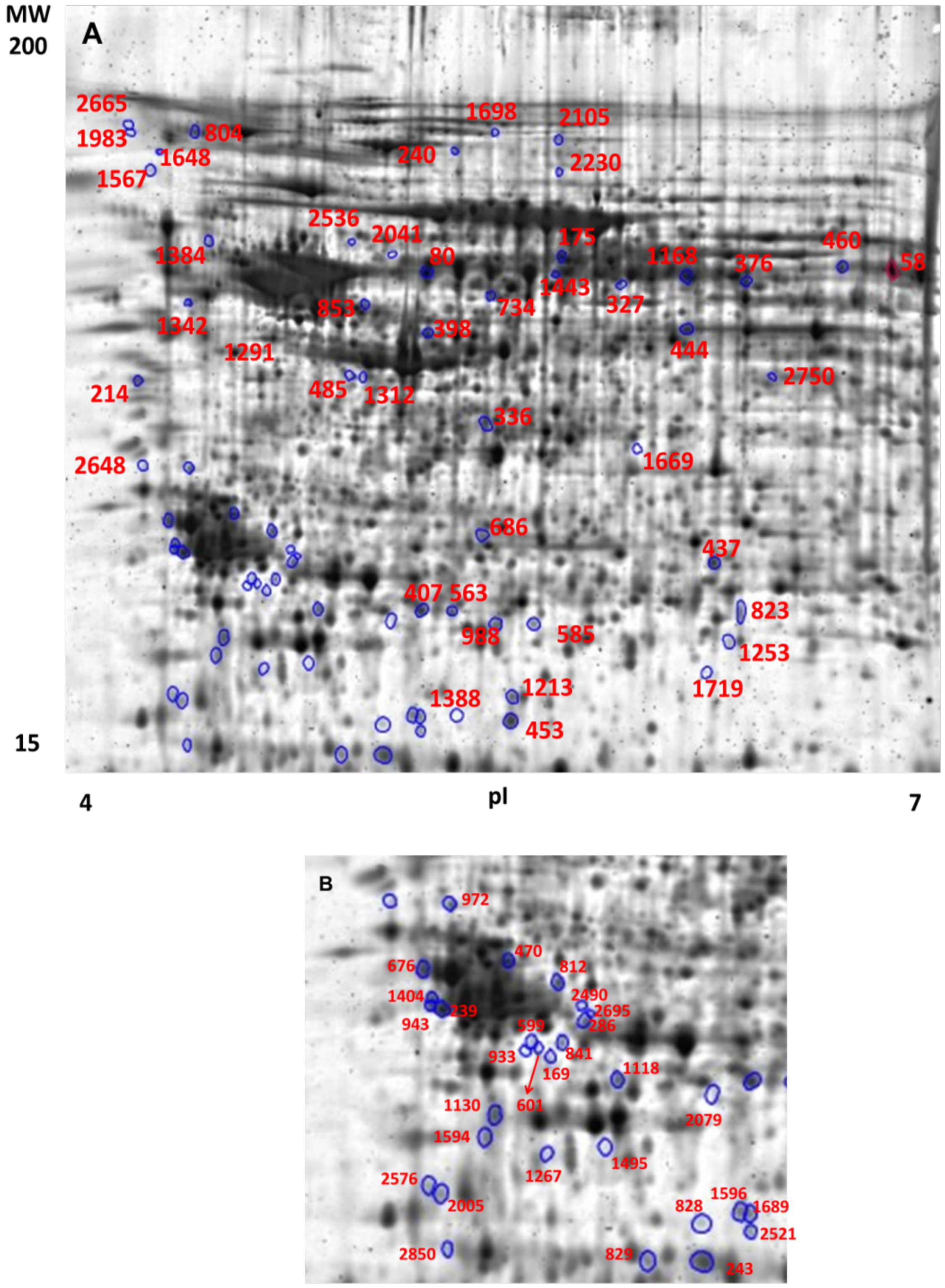
MW
200
15
A
2665
1983
804
1648
1567
1698
2105
240
2230
2536
2041
175
460
58
1384
80
1168
376
1443
853
734
327
1342
398
444
1291
2750
214
485
1312
336
2648
1669
686
437
407
563
823
988
585
1253
1719
1213
1388
453
4
pI
7
B
972
470
676
812
1404
2490
239
2695
943
286
599
933
841
169
1118
1130
601
2079
1594
1495
1267
2576
2005
1596
828
1689
2521
2850
829
243

Figure S2

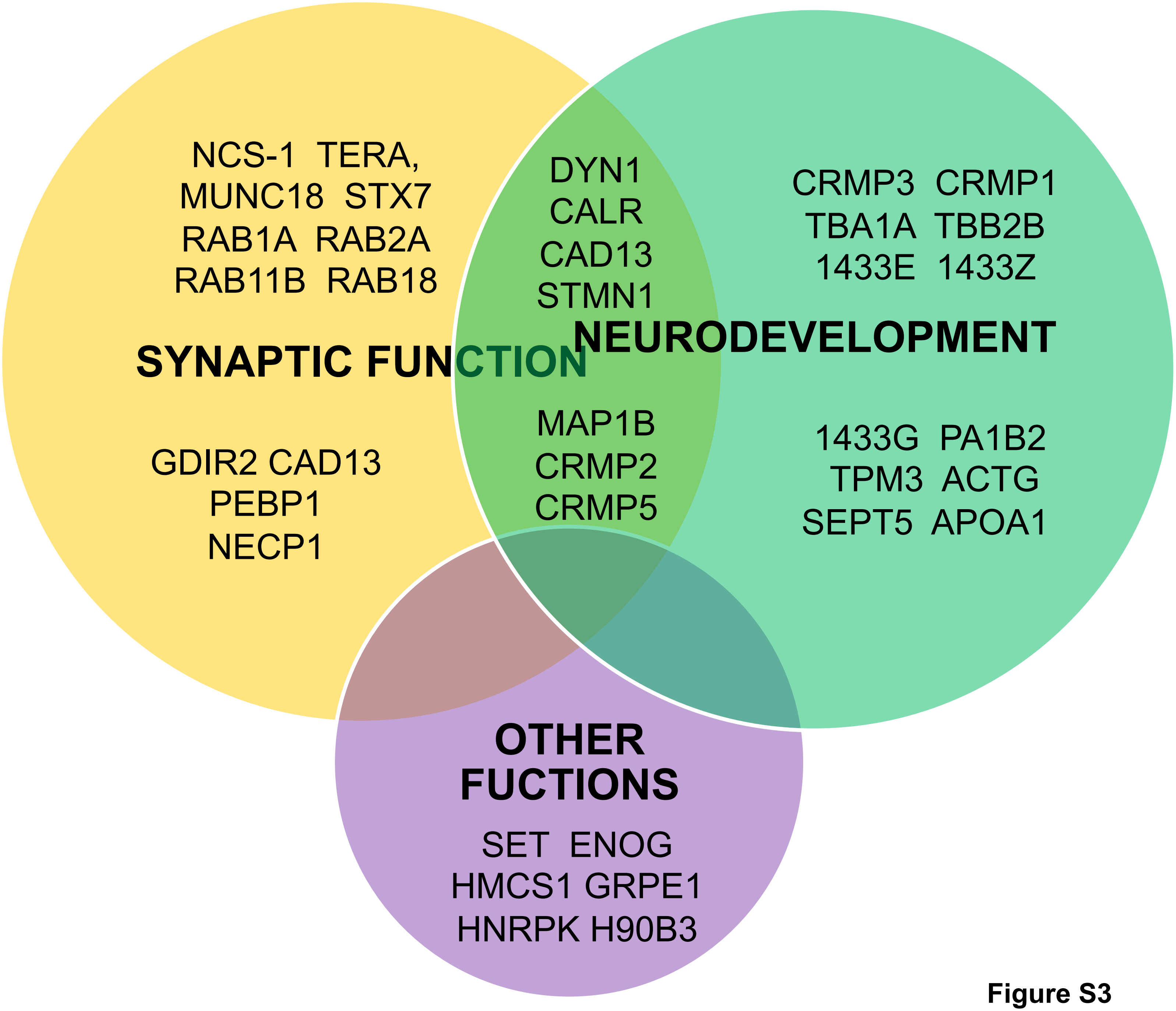
NCS-1 TERA,
MUNC18 STX7
RAB1A RAB2A
RAB11B RAB18
SYNAPTIC FUNCTION
GDIR2 CAD13
PEBP1
NECP1
DYN1
CALR
CAD13
STMN1
MAP1B
CRMP2
CRMP5
CRMP3 CRMP1
TBA1A TBB2B
1433E 1433Z
NEURODEVELOPMENT
1433G PA1B2
TPM3 ACTG
SEPT5 APOA1
OTHER
FUCTIONS
SET ENOG
HMCS1 GRPE1
HNRPK H90B3

Figure S3

Control

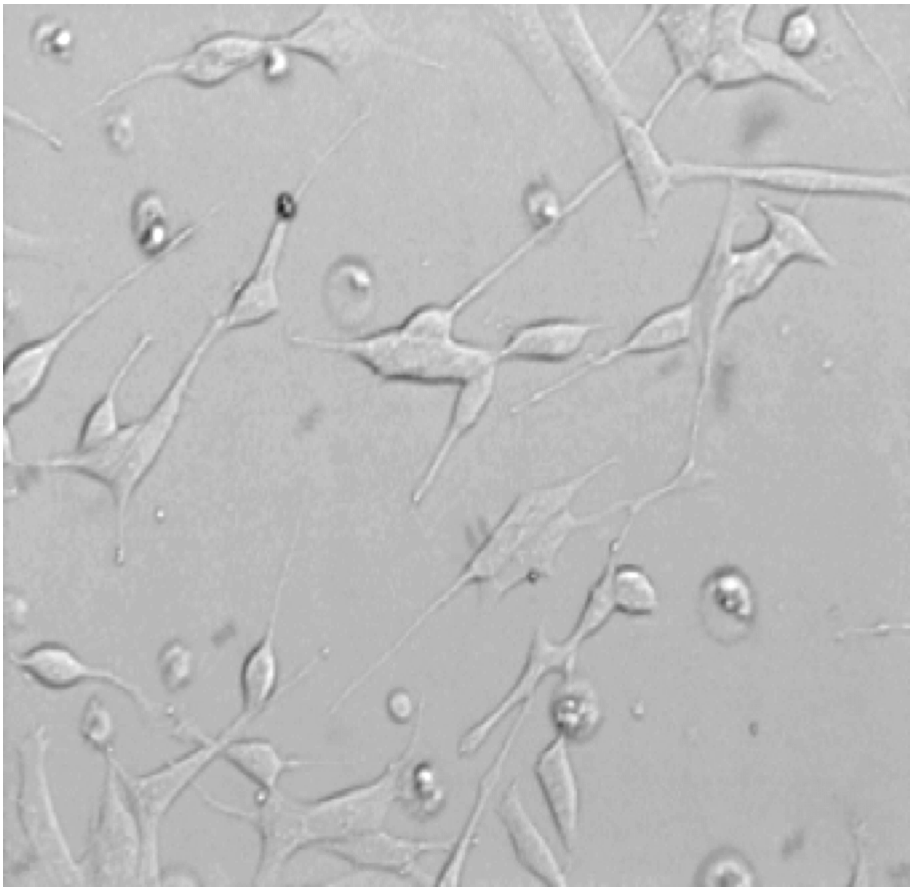

Silenced 1

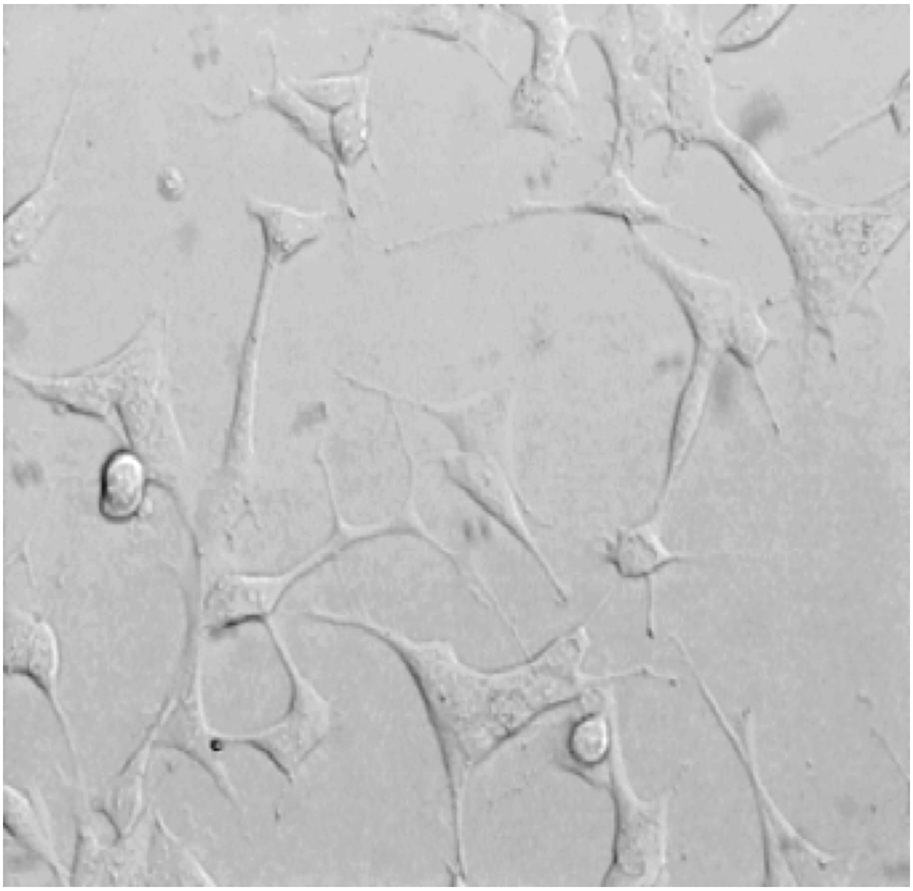

Figure S4
